# Biomarkers of inflammation in sweat after myocardial infarction

**DOI:** 10.1038/s41598-025-90240-8

**Published:** 2025-02-15

**Authors:** Anna Björkenheim, Erik Sunnefeldt, Karl Finke, Daniel Robert Smith, Ole Fröbert, Noé Brasier

**Affiliations:** 1 Department of Cardiology, School of Medical Sciences, Örebro University, 701 82 Örebro, Sweden; 2 Department III of Internal Medicine, Faculty of Medicine and University Hospital Cologne, University of Cologne, Cologne, Germany; 3 Clinical Epidemiology and Biostatistics, Faculty of Medicine and Health, School of Medical Sciences, Örebro University, 701 82 Örebro, Sweden; 4 Department of Clinical Medicine, Faculty of Health, Aarhus University, 8000 Aarhus, Denmark; 5 Department of Clinical Pharmacology, Aarhus University Hospital, 8000 Aarhus, Denmark; 6 Steno Diabetes Center Aarhus, Aarhus University Hospital, 8000 Aarhus, Denmark; 7 Department of Health Science and Technology, Institute of Translational Medicine, ETH Zurich, Zurich, Switzerland

**Keywords:** Acute myocardial infarction, Biomarkers, Inflammation, Non-invasive monitoring, Sweat analysis, Wearables, Biomarkers, Cardiology, Medical research

## Abstract

ST-elevation myocardial infarction (STEMI) triggers a significant inflammatory response. Sweat may offer a novel, non-invasive medium for monitoring inflammation. In this prospective study, we characterized the inflammatory signatures in plasma and sweat collected from the skin surface of two patient groups: (1) 18 STEMI patients immediately following percutaneous coronary intervention (exposure) and (2) six patients who underwent outpatient angiography without subsequent intervention (control). Levels of 92 biomarkers were measured using a high-throughput proteomic assay and reassessed after 4–6 weeks in STEMI patients. Adjusting for patient group, sweat biomarkers did not show significant changes over time. In plasma, hepatocyte growth factor and interleukin-6 showed a significant decrease from the acute phase to follow-up, adjusted for patient group. STAM binding protein was significantly higher in the sweat of STEMI patients compared to controls, adjusted for time effects. While sweat was less sensitive than plasma for detecting biomarker levels in the setting of STEMI, its longitudinal analysis via wearable sensors holds promise for detecting specific markers.

*Trial registration*: The trial is registered on www.clinicaltrials.gov with the trial registration number NCT05843006.

## Introduction

Acute myocardial infarction, especially transmural ischemia, triggers immediate sympathetic nervous system activation, increasing sweat rate and lowering the ventricular fibrillation threshold^1,2^. Simultaneously, a powerful transient inflammatory reaction is initiated that is essential to myocardial repair but may contribute to adverse ventricular remodeling if excessive or prolonged^3,4^. Paradoxically, reperfusion through percutaneous coronary intervention (PCI) can amplify this inflammatory response^5–8^.

Analysis of sweat is increasingly used for monitoring biochemical biomarkers in various medical conditions and is promising for heart failure management^9^. It can be collected non-invasively from the skin surface to provide valuable insight into the body’s metabolic state through its molecular signature, as well as to assess sympathetic nervous system activity through sweat rate^10–13^. Advanced wearable sensors enable the detection of metabolites, proteins, and xenobiotics, such as amino acids, high-sensitivity C-reactive protein (hs-CRP), and antibiotics, in sweat, offering new possibilities for real-time health monitoring^9,14–20^. The inflammatory signature in sweat of myocardial infarction patients remains a largely unexplored field, with potential as a non-invasive diagnostic tool for monitoring inflammatory responses.

In this pilot study, we compared the molecular inflammatory signature in the sweat and plasma of ST-elevation myocardial infarction (STEMI) patients post-PCI with that at 4–6 weeks follow-up as well as with that of controls who underwent coronary angiography for suspected angina without subsequent intervention.

## Results

### Patient baseline characteristics, laboratory analyses, and clinical outcomes

STEMI patients (n = 18, 83% male) and control patients (n = 6, 66% male) had a mean age 59 ± 6 years. The control group had a higher prevalence of previously known coronary artery disease compared to the STEMI group, with a borderline significant difference (83% vs. 28%, *p* = 0.049). Hypertension was present in 63% of all patients, and diabetes in 25%, with no significant differences between groups (Table 1).

**Table 1:** Patient characteristics and baseline laboratory findings.

	Controls (n = 6)	STEMI (n = 18)	*p* value *
Demographics			
Female sex, n (%)	2 (33)	3 (17)	0.57
Age, years, mean (± SD)	58 (5)	60 (7)	0.50
Medications baseline, n (%)			
ACEI or ARB	5 (83)	8 (44)	0.17
Aspirin	5 (83)	7 (39)	0.16
Beta blocker	6 (100)	6 (33)	**0.01**
Diuretic	2 (33)	0 (0)	0.05
MRA	1 (17)	1 (6)	0.45
Statin	6 (100)	12 (67)	0.28
Cardiovascular risk factors, n (%)			
Diabetes	2 (33)	4 (22)	0.62
Hypertension	5 (83)	10 (56)	0.35
Hyperlipidemia	5 (83)	7 (39)	0.16
Known coronary artery disease	5 (83)	5 (28)	**0.04**
Smoking			
Never	4 (67)	5 (28)	
Previous/current	2 (33)	13 (72)	0.15
Cardiovascular parameters median, (IQR)			
Systolic blood pressure, mmHg	130 (116–154)	130 (116–141)	0.72
Diastolic blood pressure, mmHg	70 (67–100)	79 (70–87)	0.54
Heart rate, bpm	65 (16)	75 (14)	0.17
Left ventricular ejection fraction (%)	55 (55–56)	50 (45–55)	0.10
GRACE score	75 (63–79)	107 (98–119)	** < 0.001**
TIMI score	1 (1–1)	1 (3–4)	** < 0.001**
Laboratory findings, median (IQR)			
Hs-CRP, mg/L	0.9 (0.2–1.4)	4.5 (1.0–8.8)	**0.03**
Leukocytes, 10 × 9/L	6.7 (5.0–7.9)	9.7 (7.5–11.5)	**0.01**
NT-proBNP, ng/L	85.5 (52.3–300.8)	654.5 (138.0–1559.3)	**0.02**
Hs-Troponin I, ng/L	7.2 (5.5–10.5)	20271.0 (10358.0–44368.3)	** < 0.001**

ACEI, Angiotensin-converting enzyme inhibitors; ARB, angiotensin receptor blockers; bpm beats per minute; GRACE, global registry of acute coronary events; Hs-TnI, high–sensitivity troponin I; Hs-CRP, high-sensitivity C-reactive protein; MRA, mineralocorticoid receptor antagonists; NT-proBNP, N-terminal pro b-type natriuretic peptide; STEMI, ST-elevation myocardial infarction; TIMI, thrombolysis in myocardial infarction.

*Statistically significant *p* values (*p* < 0.05) are marked in bold.

In STEMI patients, the median door-to-balloon time was 64 min (IQR 40.5–80.5), and the median length of hospital stay was three days (IQR 3.0–3.25). The median GRACE score for STEMI patients at PCI was 106 (IQR 84.3–115.8), and the median TIMI score was 2 (IQR 2.0–4.3).

Laboratory analyses of venous blood showed significantly higher levels of hs-TnI and hs-CRP as well as higher leukocyte counts in STEMI patients at the acute phase compared to controls (all *p* < 0.001) with decreased levels at follow-up (all *p* < 0.001) (Table 1). The median peak high-sensitivity troponin I was 41 722 (IQR 15 573–53 932) ng/L. The difference in NT-proBNP levels between STEMI patients and controls was also significant (*p* = 0.02) (Table 1). One STEMI patient suffered an aortic dissection on the day of discharge; no other cardiovascular events were reported over a 6-week follow-up period.

### Proximity extension assay

**Fig. 1:**
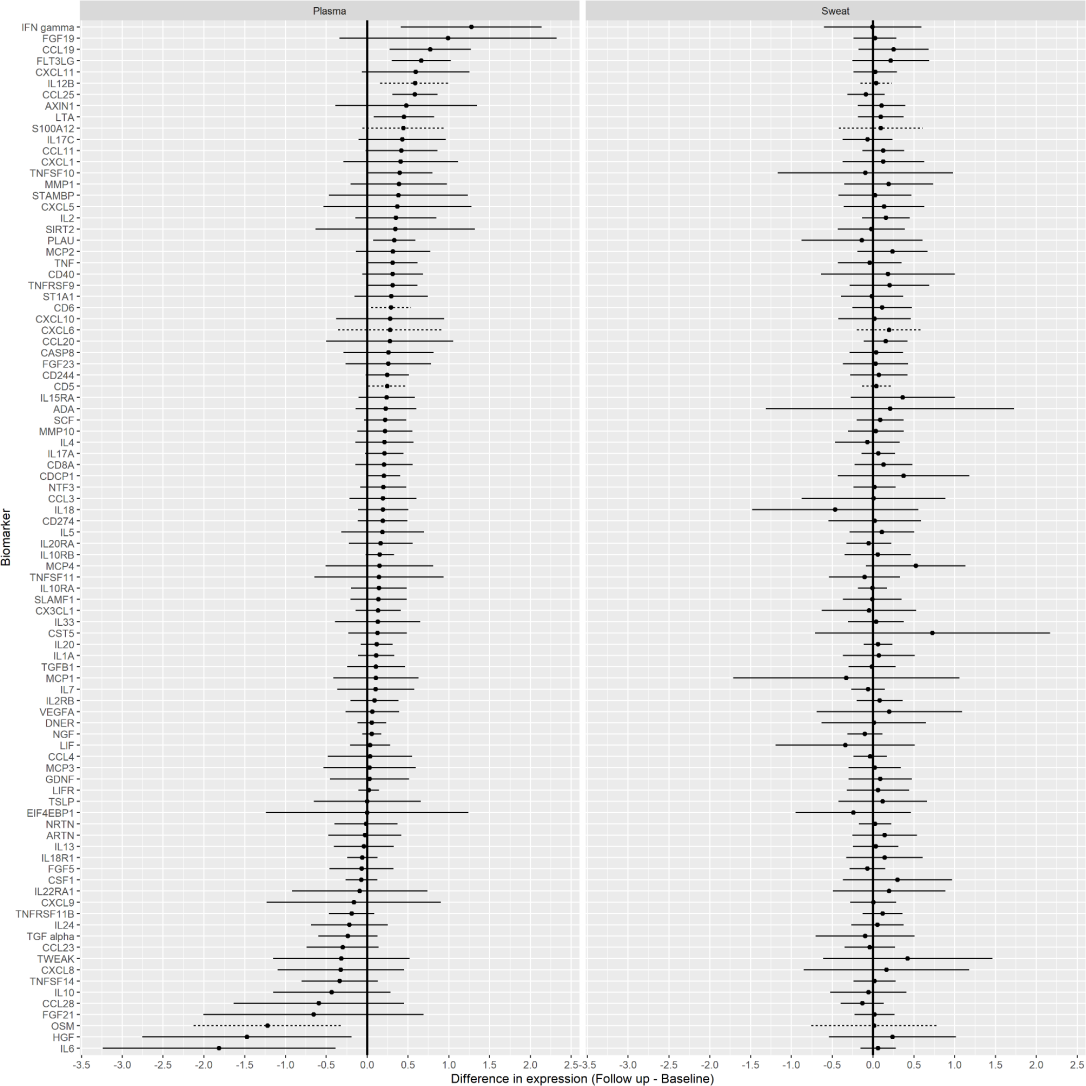
Model-based estimates of the difference in mean (acute phase–follow-up) normalized protein expression of inflammatory biomarkers in plasma and sweat of patients with ST-elevation myocardial infarction (n = 18). Biomarkers are sorted in descending order based on the magnitude of the point estimates (solid circles) for plasma. Positive differences indicate an increase in the measured biomarker level from acute phase to follow-up, while negative differences indicate a decrease. Horizontal lines extending from the point estimates denote 95% confidence intervals, adjusted for simultaneous inference. Dashed lines denote that fewer than 50% of samples were measured above the limit of detection.

Biomarkers in sweat at the acute phase and follow-up did not differ, adjusting for patient group. Ten biomarkers in plasma differed significantly between the acute phase and follow-up, adjusting for patient group (Fig. 1). Hepatocyte growth factor (HGF) and IL-6 showed a significant decrease in plasma from the acute phase to follow-up, while interferon gamma (IFN-gamma), C–C motif chemokine 19 (CCL19), Fms-related tyrosine kinase 3 ligand (FLT3LG), C–C motif chemokine 25 (CCL25), lymphotoxin-alpha (LTA), tumor necrosis factor ligand superfamily member 10 (TNFSF10), urokinase-type plasminogen activator (PLAU), and CUB domain-containing protein 1 (CDCP1) showed a significant increase from the acute phase to follow-up.

### STEMI patients versus controls

**Fig. 2:**
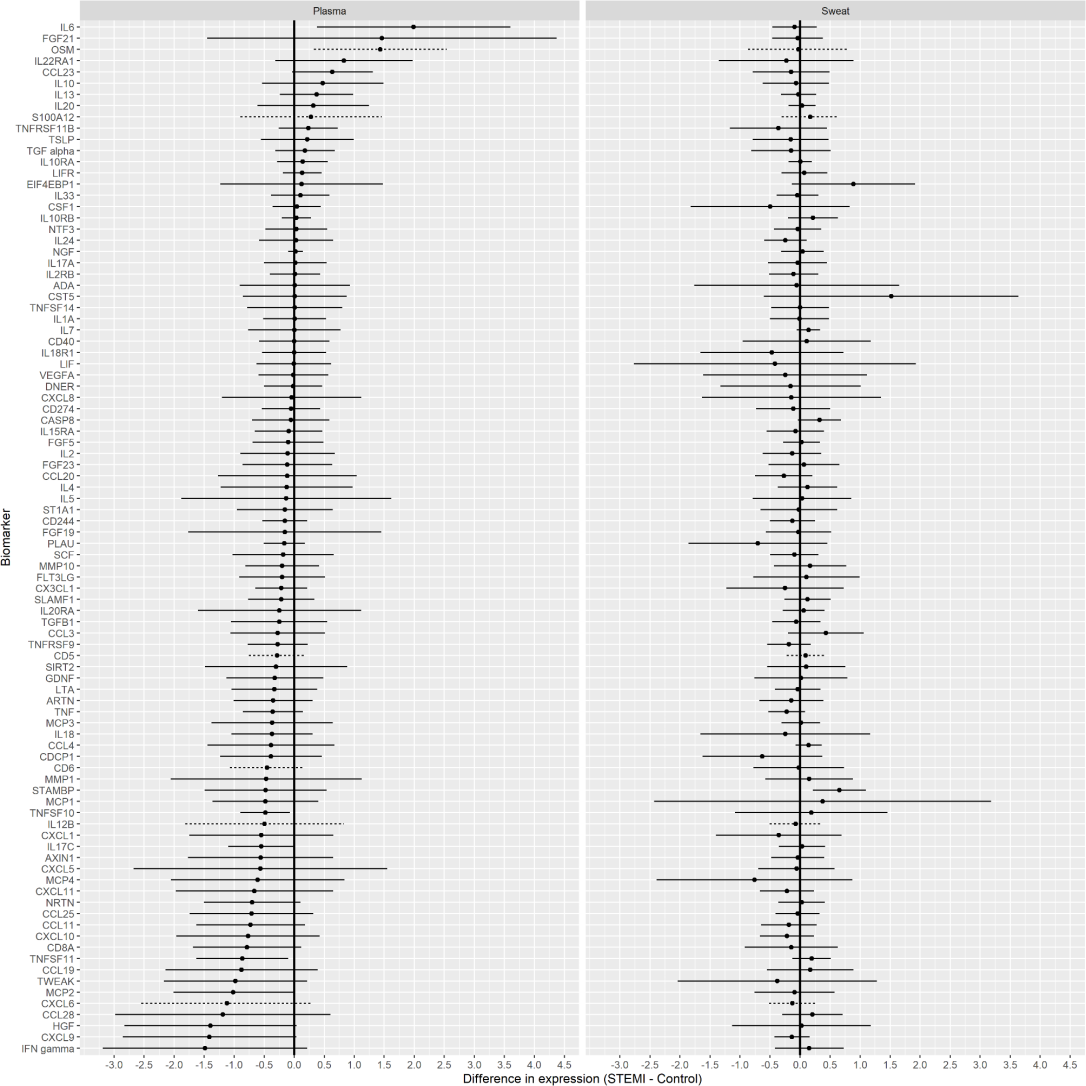
Model-based estimates of the difference in mean normalized protein expression values of inflammatory biomarkers in plasma and sweat in patients with ST-elevation myocardial infarction (n = 18) at acute phase compared to controls who underwent coronary angiography because of suspected angina without subsequent intervention (n = 6). Biomarkers are sorted in descending order based on plasma-adjusted differences. Positive differences indicate higher biomarker levels in STEMI patients than in controls, while negative differences indicate lower levels. Horizontal lines extending from the point estimates denote 95% confidence intervals, adjusted for simultaneous inference. Dashed lines denote that fewer than 50% of samples were measured above the limit of detection.

When comparing STEMI patients at the acute phase to controls, adjusted for time effects, one sweat biomarker (STAM Binding Protein, STAMBP) and four biomarkers in plasma (IL-6, TNFSF10, TNFSF11, and monocyte chemoattractant protein (MCP-2)) showed significant differences (Fig. 2). Specifically, STEMI patients had significantly higher STAMBP levels in sweat and elevated IL-6 levels in plasma compared to controls. Conversely, plasma concentrations of TNFSF10, TNFSF11, and MCP-2 levels were lower in STEMI patients relative to controls.

## Discussion

To the best of our knowledge, this study is the first to explore the proteomic inflammatory signature in the sweat of STEMI patients longitudinally, comparing these findings with plasma biomarkers and with those of stable controls. Patients with STEMI showed evidence of significant myocardial injury and systemic inflammation in standard plasma biomarker tests during the acute phase, with resolution at follow-up, as expected^3,21^. While most inflammatory panel biomarkers were detectable in both sweat and plasma, after adjusting for patient group, only a few plasma biomarkers showed significant changes over time and no significant changes were observed in sweat biomarkers. This lack of temporal variation in sweat markers suggests that sweat may not be as sensitive as plasma for detecting dynamic inflammatory changes post-STEMI, at least with respect to the selected biomarker panel. The plasma biomarkers that showed a significant decrease from the acute phase to follow-up, IL-6 and HGF, are well-established markers of inflammation and tissue repair, and their reduction over time aligns with the expected resolution of acute inflammation following myocardial infarction^22,23^.

One sweat biomarker, STAMBP, was significantly higher at the acute phase compared to controls, adjusting for patient group. STAMBP has been associated with cell processes including endosomal sorting and inflammation, but its role in cardiovascular disease is not well understood^24^. This finding suggests a potential avenue for further research into the significance of STAMBP in sweat and its clinical utility as a biomarker.

In plasma, when adjusting for time effects, IL-6 was significantly higher in STEMI patients compared to controls, which aligns with existing literature that proposes IL-6 as a key pro-inflammatory cytokine elevated during acute coronary events^25^. Interestingly, TNFSF10, TNFSF11, and MCP-2 levels were lower in STEMI patients than in controls, which may reflect the complex regulatory mechanisms involved in inflammation and immune response following myocardial infarction. TNFSF10 (also known as TRAIL) and TNFSF11 (also known as TRANCE) are known to play roles in apoptosis and regulation of the T-cell-dependent immune response^26,27^, and their lower levels in STEMI patients may reflect suppression of cell death to limit myocardial damage or depletion of susceptible cells during ischemia^28^.

Despite the promising nature of sweat for biomarker analysis using a wearable platform^9^, the results of this study suggest limitations in its capacity to reflect dynamic changes in inflammatory processes following STEMI. While detection of higher levels of STAMBP in sweat shows potential for individual markers, further research is needed to determine whether sweat can provide complementary or unique information compared to traditional plasma biomarkers in the context of acute coronary syndromes. If shown feasible, sweat analysis could help identify patients with excessive inflammation who may benefit from anti-inflammatory treatment.

A strength of this study is the use of a high-throughput proteomic platform to analyze a broad panel of inflammatory biomarkers in both sweat and plasma. The longitudinal design, with samples taken at the acute phase and at follow-up, allowed for a thorough assessment of changes over time in STEMI patients. This study is the first to explore sweat as a fluid for biomarker analysis in myocardial infarction patients, providing valuable insight into its potential utility for non-invasive monitoring.

The study has several limitations. First, the control group contained a high percentage of individuals with existing coronary artery disease, which may have influenced the biomarker profile, since atherosclerosis is a chronic inflammatory condition. The differences between STEMI patients and controls might have been more pronounced if the controls had been healthy individuals without a known history of coronary artery disease. This could have provided a clearer contrast between acute and chronic inflammatory states.

Second, samples were obtained after PCI and the initiation of antithrombotic and statin therapy, which are known to influence inflammatory biomarkers^29^, potentially masking differences between the acute phase and follow-up, as well as between STEMI patients and controls.

Third, timing of the follow-up sample may have affected the outcomes. The follow-up was conducted 6 weeks post-STEMI, a period still within the myocardial healing phase when several proteins could remain upregulated. A later follow-up could potentially provide insights into the resolution phase of inflammation and biomarker normalization. Fourth, the small sample size, the predominance of male STEMI patients in the cohort, and the exploratory nature of the study limit the generalizability of the findings. Larger studies with more diverse control groups and consideration of pre-treatment biomarker levels are needed to confirm the results.

Finally, the absence of a control group at follow-up meant that we were unable to allow the effect of patient group to vary by time using an interaction term in the regression model. However, as a pilot study, this work provides a foundation for future research. Obtaining sweat samples prior to or during PCI may provide valuable insights into acute-phase biomarker profiles, although logistics in an emergency setting can be challenging. Future studies could explore streamlined sweat-collection methods (e.g., wearable patches) upon arrival to enable a more comprehensive assessment of early biomarker dynamics.

While this study successfully characterizes the inflammatory biomarker signature in sweat following STEMI, plasma appears to be a more reliable source for demonstrating change. Further studies with larger cohorts and additional biomarkers are necessary to fully elucidate the potential role of sweat analysis in monitoring inflammation after myocardial infarction.

## Methods

### Study design and population

**Fig. 3:**
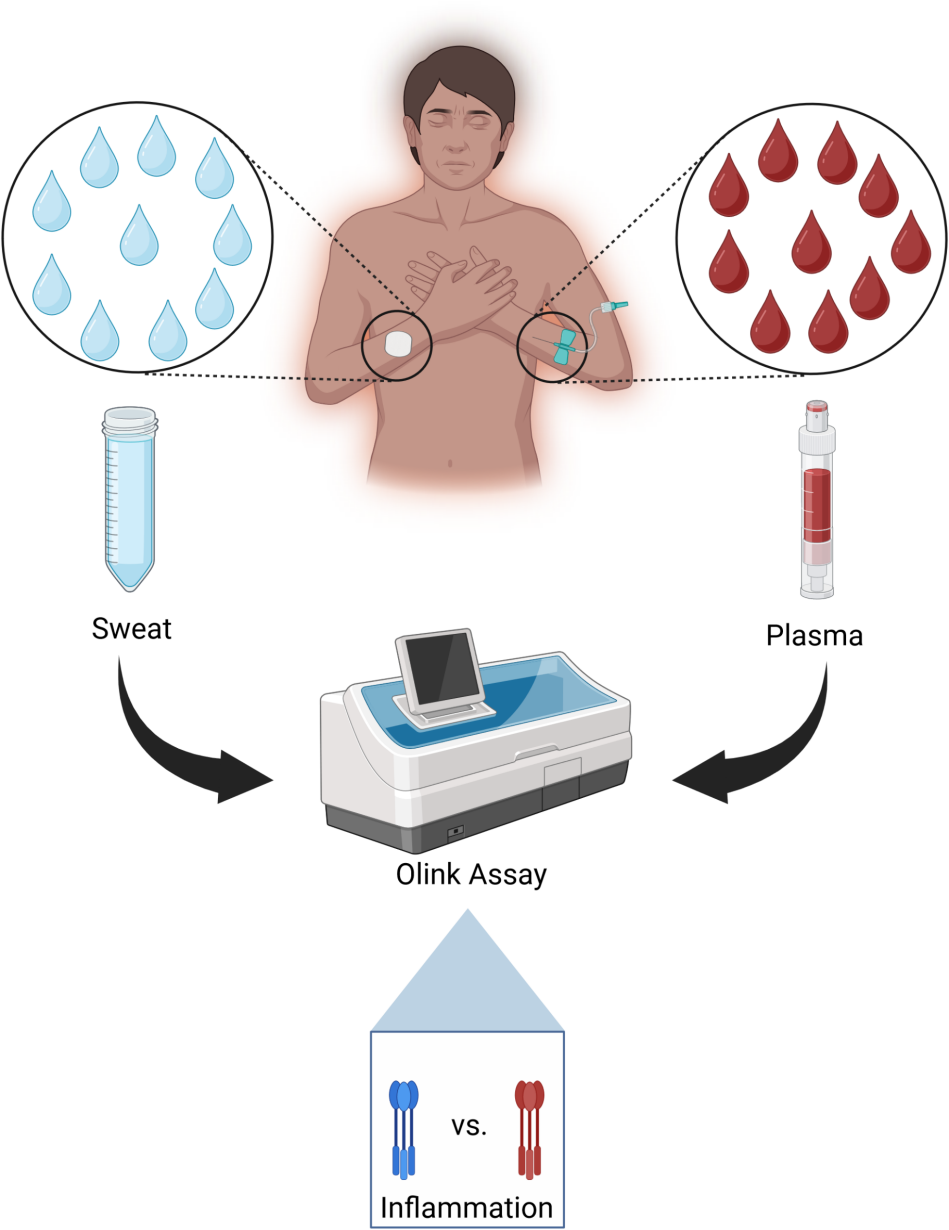
Sweat was collected non-invasively from the skin surface, and plasma was obtained from 18 STEMI patients immediately following PCI, and from 6 controls undergoing outpatient angiography without intervention. Ninety-two inflammatory biomarkers were analyzed using a high-throughput proteomic assay.

This prospective single-center observational study was conducted at the Department of Cardiology, Örebro University Hospital, Sweden. We included two groups of patients: 18 who had undergone coronary angiography with PCI for STEMI within the past 24 h and a control group (n = 6) who had undergone outpatient diagnostic coronary angiography for suspected angina with no subsequent intervention. STEMI was defined as chest pain indicative of myocardial ischemia and ECG showing new ST-segment elevation in at least two contiguous leads (≥ 2 mm in males or ≥ 1.5 mm in females for leads V2-V3, and/or ≥ 1 mm in other leads)^21,30^. Additional inclusion criteria were written informed consent and age 45–70 years. Exclusion criteria included emergency coronary artery bypass graft surgery, pacemaker treatment, immunosuppressive pharmacotherapy, and the need for supplemental oxygen. After obtaining informed consent, subjects underwent clinical data assessment as well as sweat and venous blood sampling following PCI or diagnostic coronary angiography (acute phase) (Fig. 3). Patients with STEMI also underwent sweat and blood sampling at four to 6 weeks during a routine follow-up outpatient visit.

### Objectives

The primary objective was to compare the inflammatory signature in sweat and plasma of STEMI patients immediately after PCI with that at follow-up. The secondary objective was to compare inflammatory biomarkers in sweat and plasma of STEMI patients post-PCI with those of patients who underwent coronary angiography without requiring intervention (controls) to explore the potential of sweat as a biomarker.

### Ethics compliance and trial registration

The trial was conducted in accordance with the principles of the Declaration of Helsinki and received approval from the Regional Ethical Review Board of Uppsala (2020-03556) with an amendment approved by the Swedish Ethical Review Authority (2022-05470-02). The trial was registered at ClinicalTrials.gov (NCT05843006).

### Assessment of clinical data

Clinical data, including resting heart rate and blood pressure, were collected at both the acute phase and follow-up. Left ventricular ejection fraction, assessed by echocardiography, was measured at the acute phase. The Global Registry of Acute Coronary Events (GRACE) and Thrombolysis in Myocardial Infarction (TIMI) scores for STEMI were assessed at the time of PCI to establish prognosis^31–33^. Six weeks after coronary angiography, the clinical endpoints death, new myocardial infarction, unplanned revascularization, and other cardiovascular events were assessed.

### Analysis of inflammatory biomarkers in sweat and plasma

Sweat samples were collected using the CE-certified Macroduct® Sweat Collection System (ELITechGroup, Puteaux, France). The volar forearm was disinfected with ethanol and cleaned with sterile water. Eccrine sweat glands were stimulated using pilocarpine iontophoresis with Pilogel® discs for 5 min. After cleansing the area with sterile water and drying, sweat was collected for 30 min using Macroduct® sweat collector capillary containers. The collected sweat was transferred to test tubes and stabilized with 1 mL of protease inhibitor. For plasma samples, one 6 mL EDTA tube was collected at each sampling time and centrifuged at room temperature at 2000 × g for 10 min. The EDTA plasma was aliquoted into 8 tubes (2 mL microtubes with 0.5 mL inserts) of 225 µL per tube. Both plasma and sweat tubes were frozen at − 80 °C within three hours of sampling and stored until analysis.

Biomarkers in sweat and plasma were analyzed using a proximity extension assay with the Olink® Target 96 Inflammation panel (Olink Proteomics, Uppsala, Sweden), which analyzes the levels of 92 protein biomarkers that may play key roles in inflammatory processes. Analyses were performed at the Swiss Institute of Allergy and Asthma Research, Davos Wolfgang, Switzerland. Data are presented as normalized protein expression (NPX) values, an arbitrary unit on a log^2^ scale provided by Olink Proteomics. The limit of detection was calculated separately for each assay and sample plate, estimated from negative controls included on every plate plus three standard deviations.

Venous blood was analyzed for high-sensitivity troponin-I (hs-TnI), hs-CRP, N-terminal pro b-type natriuretic peptide (NT-proBNP), and leukocyte counts at acute phase and follow-up. Analyses were conducted at the Department of Laboratory Medicine, Örebro University Hospital. High-sensitivity troponin-I and NT-proBNP were analyzed using the Siemens Centaur XPT platform; hs-CRP was measured with the Siemens ADVIA Chemistry XPT system (Siemens Healthineers, Erlangen, Germany); and leukocyte counts were determined using the Sysmex XN-9000 system (Sysmex Europe, Norderstedt, Germany), supplemented by microscopy.

### Statistical analysis

#### Statistical power analysis

A sample size of 16 STEMI patients with measurements obtained at two time points, was required to detect an effect size (Cohen’s f) of 1.5 at a significance level of 0.05 and a power of 0.8. This calculation was based on an expected decrease in interleukin-6 (IL-6) NPX values from 5 at the acute phase to 3.5 at follow-up. To account for potential drop-outs and sampling failures, two additional patients were included, bringing the total to 18 STEMI patients. Given the observational nature of the study and the exploratory focus on comparing STEMI patients and controls, six controls were deemed sufficient to maintain feasibility with respect to recruitment and resource allocation.

#### Descriptive statistics

Continuous variables are reported as mean and standard deviation or median and interquartile range (IQR) as appropriate after testing for normality using the Shapiro–Wilk test. Categorical variables are reported as frequency and percentage. Ordinal variables were evaluated using either the Chi-square test for trend or the Mann–Whitney U test. A two-sided statistical significance level of 5% was applied, with estimates presented with 95% confidence intervals, assuming an adequate sample size.

#### Data pre-processing

The NPX values were normalized using linear regression models, with residuals used in downstream analysis. For sweat samples, predictors included protease inhibitor concentration, protein concentration, and age. For plasma samples, age was the sole predictor.

#### Effect estimate

To address our research questions, we fitted two multiple linear regression models, with parameters estimated using ordinary least squares. The response variable was the normalized NPX values, and all predictors were categorical, modelled using dummy variables. For the first model, predictors included biomarker (n = 92), source (plasma, sweat), time (acute, follow-up), and patient group (STEMI, control). We allowed the effects of time and patient group to vary by each combination of biomarker and source using three-way interactions (i.e. biomarker × source × time and biomarker × source × patient group), as well as all lower-order terms. The second model, used for sensitivity analysis, was parameterized as the above but adjusted for sex, hypertension, and diabetes. These additional predictors were allowed to vary by each biomarker × source combination using three-way interactions (i.e. biomarker × source × sex; biomarker × source × hypertension; and biomarker × source × diabetes), as well as all lower-order terms. We computed a robust variance–covariance matrix of the model using the Huber-White method to correct for heteroscedasticity and correlated responses among patients. Two sets of model-based conditional contrasts of the difference in means and their 95% confidence intervals were computed for each biomarker and source combination. The first set comprised the difference between acute phase and follow-up. The second set was the difference between STEMI and controls. Confidence intervals were adjusted for simultaneous inference.

Statistical analyses were performed in R (v.4.4.0), using the packages rms^34^ and tidyverse^35^.

## Data Availability

The datasets generated during and/or analyzed during the current study are available from the corresponding author on reasonable request. The R code used to analyze differences in normalized NPX values is available from the corresponding author upon reasonable request.

## Abbreviations

CCL: C–C motif chemokine
FLT3LG: Fms-related tyrosine kinase 3 ligand
GRACE: Global registry of acute coronary events
HGF: Hepatocyte growth factor
Hs-CRP: High-sensitivity C-reactive protein
Hs-TnI: High-sensitivity troponin-I
IFN: Interferon
IL: Interleukin
IQR: Interquartile range
LTA: Lymphotoxin-alpha
MCP: Monocyte chemoattractant protein
NPX: Normalized protein expression
NT-proBNP: N-terminal pro b-type natriuretic peptide
PCI: Percutaneous coronary intervention
STAMBP: STAM binding protein
STEMI: ST-elevation myocardial infarction
TIMI: Thrombolysis in myocardial infarction
TNFSF: Tumor necrosis factor ligand superfamily member
